# Folding of newly translated membrane protein CCR5 is assisted by the chaperonin GroEL-GroES

**DOI:** 10.1038/srep17037

**Published:** 2015-11-20

**Authors:** Haixia Chi, Xiaoqiang Wang, Jiqiang Li, Hao Ren, Fang Huang

**Affiliations:** 1 State Key Laboratory of Heavy Oil Processing and Center for Bioengineering and Biotechnology, China University of Petroleum (East China), Qingdao 266580, P. R. China

## Abstract

The *in vitro* folding of newly translated human CC chemokine receptor type 5 (CCR5), which belongs to the physiologically important family of G protein-coupled receptors (GPCRs), has been studied in a cell-free system supplemented with the surfactant Brij-35. The freshly synthesized CCR5 can spontaneously fold into its biologically active state but only slowly and inefficiently. However, on addition of the GroEL-GroES molecular chaperone system, the folding of the nascent CCR5 was significantly enhanced, as was the structural stability and functional expression of the soluble form of CCR5. The chaperonin GroEL was partially effective on its own, but for maximum efficiency both the GroEL and its GroES lid were necessary. These results are direct evidence for chaperone-assisted membrane protein folding and therefore demonstrate that GroEL-GroES may be implicated in the folding of membrane proteins.

Membrane proteins synthesized on cytosolic ribosomes insert into biological membranes and fold into defined three-dimensional structures to attain functionality. Understanding how these proteins fold is not only of fundamental biological interest but also has potential for improving human health, as more than 50% of all drugs target these molecules^1^,^2^. It is generally accepted that the primary force that drives membrane integration is the overall hydrophobicity of the individual transmembrane domains of membrane proteins^3^. However, the process of folding and the factors that influence membrane insertion remain unresolved. The anisotropic lipid environment and the complex lipid composition that allow a broad spectrum of chemical and physical properties within the lipid bilayer significantly complicate the study of the folding of polytopic membrane proteins compared with water-soluble proteins^4^,^5^,^6^.

Much of our current understanding of how membrane proteins fold is based on *in vitro* studies on the functional refolding of chemically denatured proteins within membrane mimetics^7^,^8^,^9^,^10^,^11^,^12^. These experiments have provided insights into the folding of membrane proteins. However, membrane proteins are very difficult to unfold, and the extent to which unfolded states exist upon chemical denaturation remains an open question^13^,^14^,^15^. Thus, refolding experiments basically report only on the folding of partially denatured proteins into a native state. Although these studies are crucial for identifying determinants of membrane proteins folding, they give little insight into how newly translated membrane protein chains fold, especially given existing ideas that residual structure in the unfolded protein can be important for refolding, i.e. can result in a significantly accelerated folding process^16^,^17^.

Chaperonins are required for the correct folding, assembly, and translocation of newly translated polypeptide chains^18^,^19^. Most of our knowledge on chaperone assisted folding has been derived from studies of the bacterial chaperonin protein GroEL and its lid GroES (Fig. 1a), and from water soluble substrate proteins^20^,^21^,^22^,^23^,^24^,^25^,^26^,^27^. Apart from its recognized function in the translocation of membrane proteins^28^,^29^, the role of GroEL in the folding of newly translated membrane protein chains is still unclear. However, it has been demonstrated that GroEL can enhance the soluble expression or functional refolding of recombinant membrane proteins^30^,^31^. Katayama *et al.* also found that GroEL substantially inhibited aggregation during the formation of a protein transmembrane pore, probably through its hydrophobic central cavity, thereby increasing the number of the pores formed in model membranes. This is similar to chaperone assisting protein folding in the cytosol^32^. *In vitro* studies have also shown that GroEL can efficiently solubilize the functional bacteriorhodopsin (BR) membrane protein^33^. These experiments all suggest that GroEL may play a direct role in the functional folding of nascent membrane protein chains, and, just as translocons have been shown to mediate the folding of membrane proteins^34^, GroEL may be of physiological significance in membrane protein folding.

In this work, we have used a cell-free transcription-translation system to synthesize the target membrane protein, in which the folding of the newly translated polypeptide chains and the role of GroEL-GroES is directly examined. Human CC chemokine receptor type 5 (CCR5), which belongs to the physiologically important family of G protein-coupled receptors (GPCRs), was selected as the model protein. The CCR5 receptor is 352 amino acids in length, with a molecular weight of 40.5 kDa, and mediates the cellular response to inflammation as well as HIV entry into cells^35^. The recently determined crystal structure of CCR5 bound to the HIV entry inhibitor maraviroc unequivocally demonstrates the highly conserved membrane topology of GPCRs, with its seven-transmembrane α-helices connected by alternating intracellular and extracellular loops (Fig. 1b)^36^. Our results demonstrated that newly synthesized CCR5 could spontaneously fold into its biologically active state in the cell-free system, but this process was slow and inefficient. In comparison, the addition of bacterial GroEL-GroES can greatly facilitate the folding of nascent CCR5 chains by increasing the rate and yield of functional folding. The cooperation between GroEL and its lid GroES is required to more efficiently promote the folding of CCR5.

## Results

### Soluble translation of CCR5 in a cell-free system

To investigate the folding of nascent CCR5 chains and the role of the chaperonin GroEL-GroES, we first examined the cell-free translation of the receptor in its soluble form. The bacterial cell-free system used provided a coupled transcription-translation machinery similar to that in the cell, except for the lack of a cellular membrane. The open nature of this system, however, allowed us to add surfactants to solubilize the newly translated CCR5 polypeptides. The soluble translation of CCR5 was probed mainly in the presence of the non-ionic surfactant Brij-35, which is highly effective in the solubilization of GPCR membrane proteins produced via a cell-free system^37^.

**Figure 2:**
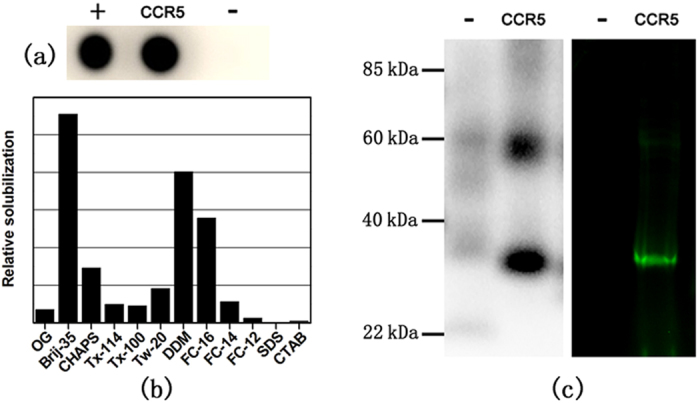
Soluble translation of CCR5 in a cell-free system. (**a**) Soluble CCR5 in the supernatant of the cell-free reaction mixture was probed via dot blot analysis. Cell-free reactions were also performed with template DNA encoding another his-tagged receptor, with CCR3, (+) and with no template DNA (−) as controls. (**b**) Surfactant screening for optimal cell-free translation of CCR5. After performing the cell-free reaction in the presence of different surfactants, samples from the reaction mixtures were first centrifuged, and supernatant protein fractions from each sample were then analyzed by dot blot, followed by spot densitometry analyses to compare the amounts of soluble CCR5. The full names of each surfactant are presented in the *Materials and Methods*. (**c**) CCR5 protein bands were analyzed by western blotting with the same negative control used in (**a**) (left) or by fluorescence imaging after the incorporation of fluorescently labeled lysine residues during the translation of CCR5 nascent chains (right). As controls, cell-free reactions were also performed without fluorescent labeling (−).

Figure 2a shows that soluble CCR5 can be synthesized in this cell-free system with the aid of Brij-35. Figure 2b shows surfactant screening results with much higher levels of soluble CCR5 expression with Brij-35 than with other commonly used surfactants, confirming the general advantages of Brij-35 in the soluble cell-free translation of GPCRs (Fig. 2b)^37^. The translated CCR5 receptor was also analyzed by Western blotting and fluorescence imaging (Fig. 2c). Two immunoreactive bands at approximately 32 kDa and 58 kDa were detected, which correspond to the monomeric and dimeric forms of CCR5. The apparent size is smaller than the theoretical molecular weight, but such discrepancy is not uncommon for GPCRs^38^,^39^. Unlike soluble proteins, which are usually boiled before loaded in SDS-PAGE gel, GPCRs cannot be boiled as boiling would cause aggregation. The incomplete denaturation by SDS alone probably resulted in a more compact shape of CCR5 and hence a faster migration^40^. As a control, fluorescent CCR5 was obtained by incorporating fluorescently labeled lysine residues into CCR5 in the cell-free expression system, and the fluorescent CCR5 bands observed via SDS-PAGE compared well with the immunoreactive bands. The yield of soluble CCR5 was estimated to be approximately 0.9 mg of receptor per mL of cell-free reaction (*Materials and Methods*), which is comparable to the yield of other GPCRs produced using the same bacterial cell-free system^41^. Taken together, these results establish that a sufficient amount of CCR5 polypeptide chains can be translated and solubilized in the cell-free reaction supplemented with Brij-35. For all subsequent experiments, Brij-35 was also included in the cell-free translation of CCR5 to improve its solubility unless otherwise stated.

### Folding of CCR5 in the presence of GroEL-GroES

**Figure 3:**
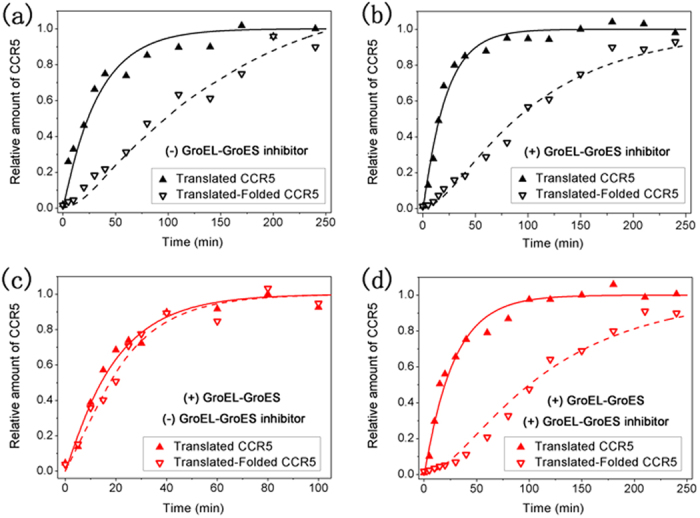
Kinetics of the translation and folding of CCR5. Representative time courses for the appearance of translated and folded CCR5 were performed in the cell-free reaction mixture, with the following additions: (**a**) none, (**b**) DMPHBA (GroEL-GroES inhibitor), (**c**) GroEL-GroES, or (**d**) GroEL-GroES and DMPHBA. The fit of the kinetic data to a simplified consecutive reaction model (*Materials and Methods*) is shown to describe the appearance of translated protein and the formation of translated-folded protein; this approach was used to estimate the apparent rate constants for these two processes.

Molecular chaperones such as GroEL-GroES are typically defined by their ability to assist the folding and assembly of proteins in a catalytic and non-consumptive manner^24^. The effect of GroEL-GroES on the folding rates of newly translated CCR5 was measured using a methodology developed by Mallam and Jackson^17^. During cell-free CCR5 synthesis, we took aliquots of the reaction mixture at various time points and halted protein synthesis by adding chloramphenicol. At this instant, fully translated CCR5 was present in both unfolded and folded states. Half of the halted reaction mixture was subjected to pulse proteolysis to digest any unfolded protein. We analyzed the undigested and digested samples by immunoblotting to monitor the translation reaction and the appearance of translated-folded CCR5 receptor, as shown in Fig. 3. It is clear that the folding rate of CCR5 is much lower than its translation rate in the absence of GroEL-GroES (Fig. 3a). The apparent rate constant for the folding of newly translated CCR5 polypeptides was estimated to be 8.2 × 10^−3^ min^−1^ based on curve fitting to a consecutive reaction model, whereas the translation rate was 3.0 × 10^−2^ min^−1^ in the absence of GroEL-GroES (*Materials and Methods*). In contrast, the addition of GroEL-GroES to the cell-free reaction significantly accelerated the folding rate of CCR5 to 0.3 min^−1^, which is approximately 36 × faster (Fig. 3c). The corresponding translation rate was approximately 5.2 × 10^−2^ min^−1^, which is a relatively small change.

To confirm the effect of GroEL-GroES on the folding of newly translated CCR5, inhibition experiments were also performed with the addition of 5-(2,5-dimethyl-pyrrol-1-yl)-2-hydroxy-benzoic acid (DMPHBA), a chemical inhibitor of the GroEL-GroES-mediated protein folding^42^, to the cell-free reaction mixture. The rates of translation and folding of CCR5 were determined to be respectively 4.6 × 10^−2^ min^−1^ and 9.6 × 10^−3^ min^−1^ after the addition of DMPHBA (Fig. 3b), which are comparable to the rates measured in the absence of DMPHBA (Fig. 3a). This result means that the amount of intrinsic GroEL-GroES in this cell-free system, if any, and its effect on the folding of CCR5 are negligible. Remarkably, while the addition of GroEL-GroES significantly accelerated CCR5 folding as indicated above (Fig. 3c), the folding rate became considerably slower with the addition of both the chaperonin complex and DMPHBA (a value of 9.1 × 10^−3^ min^−1^ was estimated from the data) (Fig. 3d). Moreover, in the latter case, both the folding rate and the translation rate (3.6 × 10^−2^ min^−1^) compared well with the values determined before the addition of GroEL-GroES, indicating full inhibition of the added chaperonin complex by DMPHBA. Taken together, the results of kinetics of translation and folding for *in vitro*-translated CCR5 clearly suggest that GroEL-GroES plays an important role in CCR5 folding and can significantly increase the rate and efficiency of folding.

**Figure 4:**
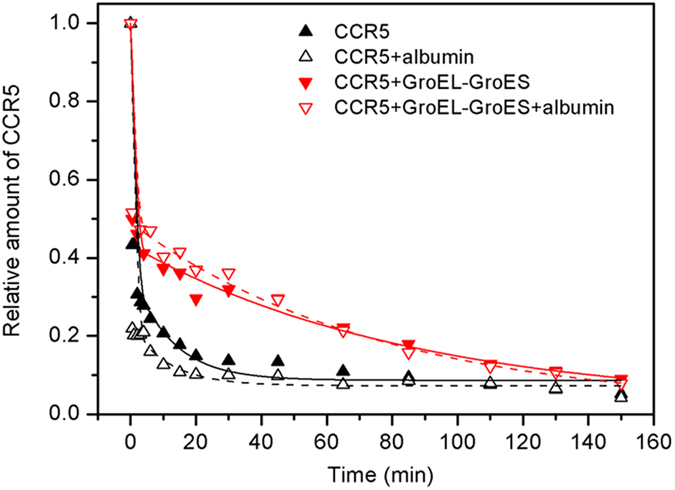
Kinetics of CCR5 proteolysis by subtilisin. Representative time courses for the proteolysis of CCR5 produced with and without GroEL-GroES added to the cell-free reaction were compared. Proteolytic reactions supplemented with bovine albumin were also performed as controls. The molar concentration of albumin was the same as the concentration of the added GroEL-GroES. The fit of the kinetic data to a 2-phase exponential equation (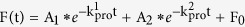) is shown to describe the different CCR5 proteolytic processes.

Proteins in well-folded conformations usually show higher resistance to proteolysis than their unfolded counterparts^43^. The kinetics of proteolysis therefore reflects the folding status of the target protein. We evaluated the structural stability of CCR5 produced with and without the addition of GroEL-GroES by measuring the kinetics of CCR5 proteolysis by subtilisin (Fig. 4). Proteolysis appeared to comprise two digestion events that occurred on very different time scales. The fast digestion process was completed within a few minutes. The relative protein amounts at ~0 min were estimated to be 0.43 and 0.50 for CCR5 synthesized without and with the addition of GroEL-GroES, respectively, and these values changed to be 0.22 and 0.52 with supplied albumin (Fig. 4). The fast process can be attributed to unfolded CCR5 polypeptides that are highly susceptible to subtilisin proteolysis. The slow process, however, required more than 100 min, depending on the folding status of CCR5. The slow phase can be attributed to folded CCR5, which has a much higher resistance to digestion by subtilisin. The proteolysis curves could be fitted well with a two-exponential equation (Fig. 4). Although the digestion rate for the fast phase could be obtained from the exponential fit, yielding rate constants ranging from 2.3 to 3.9 min^−1^, these values only provided an approximate order of magnitude because this phase was too fast to be accurately determined. The proteolysis rate constant for the second phase was also obtained from curve fitting. As shown in Table 1, the rate constants were 4.3 × 10^−2^ min^−1^, 2.3 × 10^−2^ min^−1^ and 1.2 × 10^−2^ min^−1^ for CCR5 synthesized without the addition of chaperone, with the addition of GroEL only and with the addition of GroEL-GroES, respectively. These results show that the presence of GroEL alone can decrease the digestion rate by approximately 2× and that the addition of GroEL-GroES can further decrease the digestion rate by almost 4×. Clearly, chaperones can facilitate the folding of CCR5 into a structure with a higher resistance to subtilisin. Figure 4 also shows that the relative amplitude of the slow phase is also distinct, *i.e.*, with the addition of GroEL-GroES, the amplitude for the slow phase is much higher, increasing from 0.22 to 0.45 for A_2_/(A_1_ + A_2_), where A_1_ is the amplitude for the fast phase and A_2_ the amplitude for the slow phase. The small relative amplitude for the case without chaperones suggests that the efficiency is very low when CCR5 folds without the assistance of chaperones, although it can fold spontaneously, whereas more folded CCR5 can be obtained in the presence of GroEL-GroES. Control experiments were carried out with the addition of albumin. In these experiments, the proteolysis rate did not change noticeably, which suggested that the decreased proteolytic rate in the presence of chaperonin was not due to increased substrate concentration (Fig. 4).

**Figure 5:**
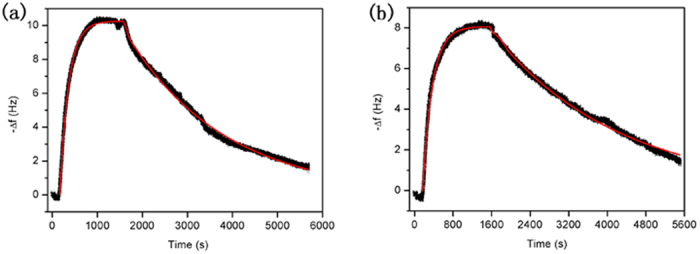
QCM sensorgrams for the binding of CCR5 to its ligand, eotaxin. Representative time-courses with visible association and dissociation phases are shown for CCR5 samples produced in the absence (**a**) or presence (**b**) of added GroEL-GroES. The fit of the kinetic data to a 1:1 binding model is shown to obtain k_a_, k_d_ and K_D_ values.

To compare the folding status of CCR5 in the presence and absence of chaperonins, the ligand binding activity of CCR5 was measured. The binding interactions between the receptor and its ligand, eotaxin (CCL11)^44^, were evaluated using a quartz crystal microbalance (QCM), and the results are shown in Fig. 5. A typical time-course comprising association and dissociation phases is observed, indicating that the CCR5 receptors obtained are biologically active regardless of the addition of GroEL-GroES. The dissociation equilibrium constant (K_D_), as assessed by fitting the kinetic data to a 1:1 binding model^45^, was estimated to be 9.7 × 10^−8^ M for CCR5 produced in the absence of GroEL-GroES. The K_D_ decreased by 2× to 4.4 × 10^−8^ M when GroEL-GroES was added to the cell-free synthesis system. These values, which are independent of the total amounts of functional receptor analyzed, are in reasonable agreement with those measured previously, as well as those for another chemokine receptor, CCR3^45^,^46^. The results of our ligand binding measurements suggest that nascent CCR5 chains can spontaneously fold into their native state in the solubilizing agent Brij-35. However, this process is very inefficient in the absence of GroEL-GroES, with a low folding rate and yield, as well as a reduction in binding affinity and structural stability. All of these results suggest that the added chaperonin complex can promote the folding of newly translated CCR5.

Apart from the folding rate and binding affinity, the addition of GroEL-GroES to the cell-free system also improved the production of soluble CCR5. The addition of GroEL-GroES increased the yield of CCR5 production from ~0.9 mg/ml to ~1.2 mg/ml. The chaperonin complex is unlikely to affect the rate or efficiency of transcription and translation. Instead, the increased production of soluble CCR5 is probable due to the more efficient folding and improved solubility of CCR5.

### The role of GroES in CCR5 folding

The essential chaperonin GroEL typically works with its lid GroES to mediate the folding of substrate proteins. However, in some cases, GroEL alone can be sufficient to assist the folding of proteins without the cooperation of GroES^47^,^48^. We therefore also examined the effect of GroEL alone on the expression, folding kinetics, structural stability and biological activity of soluble CCR5 to assess the role of GroES in the folding of newly translated CCR5.

As shown in Supplementary Fig. 1a, the level of soluble CCR5 expressed with the aid of the surfactant Brij-35 did not appear to increase after the addition of GroEL alone to the cell-free system. As expected, the lid chaperonin GroES alone also had no influence on the translation of soluble CCR5. Nevertheless, the addition of GroEL alone accelerated the formation of folded receptor (Supplementary Fig. 1b and Table 1). The rate constant for CCR5 folding in the presence of added GroEL alone was estimated to be 7.0 × 10^−2^ min^−1^, which is approximately 8× faster than without the addition of chaperonin but slower than that with the complete GroEL-GroES complex. In terms of the structural stability and ligand-binding capacity of CCR5, the addition of GroEL alone to the synthesis process also exerted a noticeable effect (Supplementary Fig. 1c and 1d). As shown in Table 1, the slower phase of proteolysis decreased by approximately 2× to 2.3 × 10^−2^ min^−1^ for CCR5 synthesized with the addition of GroEL alone. The dissociation equilibrium constant also decreased from 9.7 × 10^−8^ M to 5.5 × 10^−8^ M when GroEL was added. However, the proteolytic rate (1.2 × 10^−2^ min^−1^) and the K_D_ (4.4 × 10^−8^ M) for CCR5 produced in the presence of GroEL-GroES suggest that the presence of GroES can further promote the folding of CCR5. These values also indicate the importance of the cooperation of GroEL and GroES.

## Discussion

CCR5 polypeptides can be translated and solubilized in the cell-free system supplemented with Brij-35. The *in vitro* synthesis of soluble CCR5 with the aid of Brij-35 facilitates the subsequent characterization of the folding reaction of nascent CCR5 chains in aqueous solution and the effect of GroEL-GroES on this process. The interactions between surfactants and the chaperonins have been studied previously. At high SDS concentrations, more than 0.8 mM, the essential chaperonin GroEL was shown to maintain its native conformation^49^. It was also demonstrated by Goulhen *et al.* that the non-ionic surfactant n-octyl-polyoxyethylene used at a concentration of 0.3% (w/v) had little effect on chaperonin-assisted refolding of the target membrane protein^30^. Brij-35, also known as n-dodecyl polyoxyethylene, is very similar in structure to n-octyl-polyoxyethylene. Given the previous studies it is believed that the non-ionic Brij-35 surfactant used at a concentration of 0.2% (w/v) in this work would have little effect on the chaperonin activity.

Without the addition of GroEL-GroES to the cell-free system, the half-life for the folding of newly translated CCR5 was determined to be approximately 85 min. This process is considerably slower than the folding of normal single-domain proteins in cells^50^. It is also approximately 10-fold slower than the *in vitro* folding of chemically denatured BR, a GPCR-like bacterial seven-transmembrane receptor, whose folding rate is ~8.4 × 10^−2^ min^−1 ^12. The much faster folding of BR can be explained by the fact that BR is not fully unfolded even at high SDS concentrations^14^,^15^,^51^. Residual structures in the unfolded state are considered to be important for protein refolding and can make a substantial contribution to the faster refolding of a chemically denatured protein compared with its newly translated counterpart^17^,^52^. Given the initial folding state of the nascent CCR5 chain and the environmental dependency of protein folding^12^,^53^, the folding rate of CCR5 determined here is reasonable. However, such a slow spontaneous folding process does not appear to be functionally beneficial, and much faster folding is expected *in vivo*.

The ligand binding measurements show that the receptor obtained in the absence of chaperones was biologically functional. This suggests that nascent CCR5 chains can spontaneously fold into their native state after being solubilized in Brij-35 micelles, which parallels the structural adaptation of membrane proteins to the phospholipid bilayer *in vivo*^6^. Although the non-ionic surfactant Brij-35 shows general advantages for the soluble cell-free expression of GPCR membrane proteins, its chemical structure and micellar aggregate, though not its amphiphilic character, are different from a bilayer. Folding in the phospholipid bilayer could therefore also be different.

In comparison, the added GroEL-GroES chaperonin complex significantly promoted the efficiency and kinetics of the folding of newly translated CCR5. With the addition of GroEL-GroES, the apparent rate constant increased 36×, requiring approximately 3 minutes to fold, which is much more reasonable for CCR5 to become functional. It was also observed that the processes of CCR5 proteolysis appeared to comprise two different enzymatic digestion events. The rate of the faster digestion process is not affected by the addition of GroEL-GroES, but the slower phase is notably different. Given that the folded states are the same with and without the addition of GroEL-GroES, proteolysis would be expected to be the same for the slow phase. The higher proteolysis rate for CCR5 produced without the addition of GroEL-GroES indicates that the folded states are different in the absence of GroEL-GroES. Without the assistance of chaperonins, the protein might not have fully folded into its native state or may have been present as a mixture of properly folded CCR5 and partially folded or misfolded states. This observation is consistent with the ligand-binding results, where CCR5 produced without the addition of GroEL-GroES displayed a lower binding affinity, assuming that some folding intermediates or partially folded CCR5 also bind the ligand but with a weaker affinity than that of the native receptor.

The existence of the fast phase of proteolysis suggests that whether or not the chaperonin complex was added, unfolded CCR5 polypeptides were present in the cell-free protein synthesis system. Nevertheless, these experiments indicate that nascent CCR5 chains can spontaneously fold without the assistance of GroEL-GroES, which is consistent with the results of the CCR5 ligand-binding experiments. However, the relative amplitude of the slow phase, which corresponds to the relative amount of folded CCR5, is noticeably different and is much greater in the presence of GroEL-GroES. This suggests that GroEL-GroES can significantly improve the folding efficiency of CCR5. However, unfolded CCR5 was still present even with the addition of GroEL-GroES, which corresponds to the amplitude of the fast phase of proteolysis, although the relative amount was considerably decreased. Given that CCR5 can fold more efficiently *in vivo*, other chaperones could participate in the folding process.

**Figure 6:**
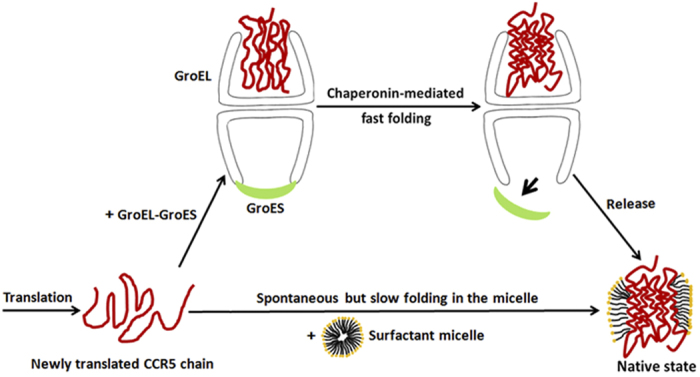
A model of GroEL-GroES action during the folding of newly translated CCR5.

Although CCR5 can fold spontaneously, this process was slow and inefficient. With the assistance of GroEL-GroES, folding was markedly accelerated and more efficient. It is reasonable to expect that during the spontaneous folding of CCR5 in Brij-35 micelles, the protein could become trapped in misfolded sates. However, with the assistance of GroEL-GroES, the folding free energy landscape becomes smoother, such that the protein is not trapped, resulting in a more rapid folding process (Fig. 6). Previous studies have highlighted the importance of hydrophobic surfaces of non-native substrate proteins in the recognition by GroEL^20^,^48^. The hydrophobic interaction between the cavity wall of GroEL and CCR5 polypeptide is also expected to play an important role in this process, as well as in affecting folding efficiency^54^.

Taken together, the results suggest that GroEL-GroES can efficiently promote the functional folding of newly translated CCR5 by increasing the rate and efficiency of folding, as well as the final yield of soluble product. We have also demonstrated that the folding of newly translated CCR5 can be promoted to some extent by the addition of GroEL alone. This result confirms the essential role of GroEL in mediating protein folding^22^,^24^. However, when compared with the addition of the complete GroEL-GroES complex, our results establish that the presence of GroES is necessary for the essential chaperonin GroEL to promote the much more efficient folding of CCR5. Given the crowded and complex interior environment of cells, which is inherently hostile to the productive folding of aggregation-prone proteins^55^,^56^, these results also raise the possibility that protein chaperones, in addition to natural lipids, can play an indispensable role in the folding of membrane protein on relevant timescales.

The role of GroEL-GroES in the folding of water-soluble proteins is well known, and based on the size of substrate proteins, two types of classical active mechanisms have been proposed for the chaperonin complex, namely, a *cis* mechanism (for substrates with molecular weights <60 kDa) or a *trans* mechanism (>60 kDa)^23^,^24^. Although GPCRs are less than 60 kDa, the hydrophobic character of their native states seems to prefer a *trans* mechanism (Fig. 6)^21^,^23^. Besides, Tehver *et al.* proposed a kinetic model for chaperonin-assisted folding that takes into account the coupling between substrate protein folding, GroEL allostery, and a pathway leading to substrate protein aggregation^57^. This multi-timescale model was shown to agree well with experimental data; meanwhile, optimized chaperonin activity was shown to depend not only on the timescales in the reaction cycle of GroEL but also on the aggregation and folding characteristics of substrate proteins. The seven-transmembrane CCR5 is highly aggregation-prone and has distinct folding behaviors compared with water-soluble proteins, which probably affected chaperonin function significantly in this study. Thus the multi-timescale kinetic framework might explain why with the assistance of GroEL-GroES folding time of CCR5 (~3 min) is an order of magnitude longer than that reported for water-soluble proteins (~10 s)^20^,^22^. A better understanding of the difference in CCR5 folding in the presence and absence of GroEL-GroES requires further investigation.

In summary, we gained important new information about the folding of the membrane protein CCR5 and, in particular, the role of the GroEL-GroES chaperone system in this process. The results also reveal a new role for GroEL-GroES towards membrane proteins, which highlights the mechanistic flexibility and substrate diversity of GroEL-GroES.

## Materials and Methods

### Cell-free CCR5 translation

The Expressway^TM^ Maxi cell-free *E. coli* system (Invitrogen) was used for *in vitro* translation of CCR5. Template plasmid DNA (1 μg) encoding C-terminally 6 × His-tagged CCR5 inserted into a pIVEX2.3d vector (Roche Diagnostics) was added to the cell-free protein synthesis reaction (100 μl, 1.2 mM ATP) and incubated at 33 °C for 3 h. The His tag fused to CCR5 was used for protein detection or immobilization during protein function analysis. Detergents or molecular chaperones were added to the cell-free reaction as required. After *in vitro* protein synthesis, samples of the reaction mixture were centrifuged, and the supernatant protein fraction of each sample was analyzed by immunoblotting or SDS-PAGE to detect soluble CCR5. The yield of soluble CCR5 in Brij-35 was estimated by densitometric quantification after immunoblotting using a standard curve generated from a purified His-tagged GPCR of known concentration (Supplementary Fig. 2). Protein synthesis was also carried out in the presence of BODIPY-Lys-tRNA_lys_ (FluoroTec GreenLys *in vitro* Translation Labeling System; Promega) to incorporate fluorescently labeled lysine residues into nascent CCR5 chains during translation.

### Western blotting, dot blotting and SDS-PAGE of fluorescently labeled CCR5 chain

For western blotting, samples were first prepared and loaded onto a Novex 10% Bis-Tris SDS-PAGE gel (Invitrogen) according to standard protocols, except that the samples were incubated at room temperature prior to loading because boiling caused membrane protein aggregation. After the samples were resolved on an SDS-PAGE gel (run in NuPAGE MOPS buffer at 100 V), they were subsequently transferred to a 0.45 μm nitrocellulose membrane. The nitrocellulose was probed with HRP-linked mouse anti-His tag antibody (TIANGEN), followed by detection using the SuperSignal West Pico kit (Thermo Scientific). For dot blots, 3 μl of each sample was directly pipetted onto a 0.45 μm nitrocellulose membrane. The samples were allowed to air dry for 30 min and were then subjected to antibody binding and detection as for western blotting. For SDS-PAGE of fluorescently labeled CCR5 chains, cell-free reaction products without boiling were directly resolved on an SDS-PAGE gel. All immunoblotting and fluorescence images were captured using an FLA-5100 imaging system (Fujifilm).

### Detergent evaluation

For the optimal soluble translation of CCR5, twelve detergents were chosen and evaluated based on their efficacy in previous membrane protein studies, including n-octyl-β-D-glucoside (OG), PEG-(23)-lauryl ether (Brij-35), 3-[(3-cholamidopropyl)-dimethylammonio]-1-propanesulfonate (CHAPS), PEG-tert-octylphenyl ether (Tx-114), PEG-p-(1,1,3,3-tetramethylbutyl)-phenyl ether (Tx-100), PEG-(20)-sorbitan monolaurate (Tw-20), n-Dodecyl-β-D-maltoside (DDM), n-dodecylphosphocholine (FC-12), n-tetradecylphosphocholine (FC-14), n-hexadecylphosphocholine (FC-16), sodium dodecyl sulfate (SDS) and cetyl trimethylammonium bromide (CTAB). Detergents were added directly in the cell-free reaction at a concentration of 0.2% (w/v), which is well above their specific critical micelle concentration. After protein synthesis, samples of the reaction mixture were centrifuged, and the supernatant protein fraction of each sample was analyzed by dot blotting. The relative amounts of soluble CCR5 in the presence of different detergents were then quantified by spot densitometry.

### Translation and folding kinetics

The time-course for the appearance of newly translated, folded CCR5 receptors was measured using the method of Mallam and Jackson^17^. Aliquots were collected at various time points after the cell-free protein synthesis reaction was initiated at 33 °C by the addition of template plasmid DNA, and translation was halted by the addition of chloramphenicol to a final concentration of 2 mM. Half of this quenched translation reaction was immediately subjected to a 1-min pulse proteolysis by subtilisin (Sigma-Aldrich) at a concentration of 7.4 × 10^−2^ μM to digest any full-length translated protein that remained unfolded. Both undigested and digested samples were analyzed by immunoblotting against the 6 × His tag, and the relative amount of CCR5 was quantified by densitometry and then normalized to its highest value for each process to compare the time-course for the appearance of full-length translated protein to that of the full-length folded protein. These measurements were repeated under identical translation conditions after the addition of the GroEL-GroES inhibitor 5-(2,5-dimethyl-pyrrol-1-yl)-2-hydroxy-benzoic acid (DMPHBA, 200 μM; Key Organics), GroEL-GroES complex (0.04 (tetradecamer): 0.08 (heptamer) μM; ProSpec), GroEL alone (0.04 (tetradecamer) μM), or GroEL-GroES complex and DMPHBA. We adopted a consecutive elementary reaction model




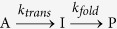




to describe the translation and folding kinetics of CCR5^58^. A, I and P represent the reactant, intermediate (translated-unfolded protein) and product (folded protein), respectively. *k*_*trans*_ and *k*_*fold*_ are the rate constants of translation and folding, respectively. This greatly simplified approach to modeling the kinetic data makes it easier to estimate the rate constants to describe the appearance of full-length translated CCR5 and full-length folded receptor. The rate equations were given by




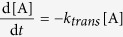







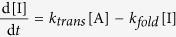







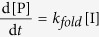




and the real-time relative concentrations of A, I and P can be represented as follows:




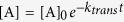







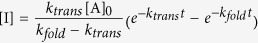







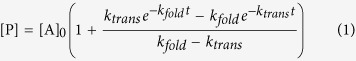




The concentration of the translated protein can be given by




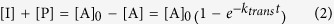




The rate constant of translation can be obtained by fitting the kinetic data using Eq. 2. The rate constant of folding can also be evaluated by substituting *k*_*trans*_ into Eq. 1. The fitted rate parameters can be found in Table 1.

### Proteolysis kinetics

CCR5 proteolysis was initiated by adding subtilisin directly to the reaction mixture after protein synthesis, at a final concentration of 5.6 × 10^−2^ μM, and the reaction was placed at room temperature (20–22 °C). Aliquots were taken at various time points, and proteolysis was halted by the addition of EDTA to a final concentration of 10 mM. The digested samples were analyzed by immunoblotting to determine the proteolytic susceptibility of CCR5 synthesized in the absence or presence of the added chaperonins. The concentrations of chaperonin used were the same as in the translation and folding kinetic study. As controls, proteolytic reactions supplemented with bovine albumin were also performed. The final concentration of albumin was the same as that of the added chaperonins. The kinetic data can be fitted using the following 2-phase exponential equation:




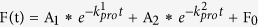




### Ligand-binding assay

The binding interaction between CCR5 and its ligand eotaxin (CCL11) was studied by quartz crystal microbalance (QCM) using a Q-Sense His-tag Capturing Sensor at 25 °C. The running buffer was HEPES buffer (25 mM HEPES, 0.12 M NaCl, 0.2% (wt/vol) Brij35, pH 7.6). After *in vitro* protein synthesis, samples of the reaction mixture were centrifuged, and the supernatant protein fraction of each sample was diluted 20 times and immobilized on the sensor. CCL11 without a His tag was expressed and purified in our lab. The binding of ligands to CCR5 immobilized on the sensor was monitored in real time, with the mobile phase flowing at a rate of 50 ml/min. As a control, the binding between CCR5 and bovine albumin was also monitored. The final concentration of albumin was the same as that of CCL11 (1 μM) (Supplementary Fig. 3).

## Additional Information

**How to cite this article**: Chi, H. *et al.* Folding of newly translated membrane protein CCR5 is assisted by the chaperonin GroEL-GroES. *Sci. Rep.* **5**, 17037; doi: 10.1038/srep17037 (2015).

## Supplementary Material

Supplementary Information

## Figures and Tables

**Figure 1:**
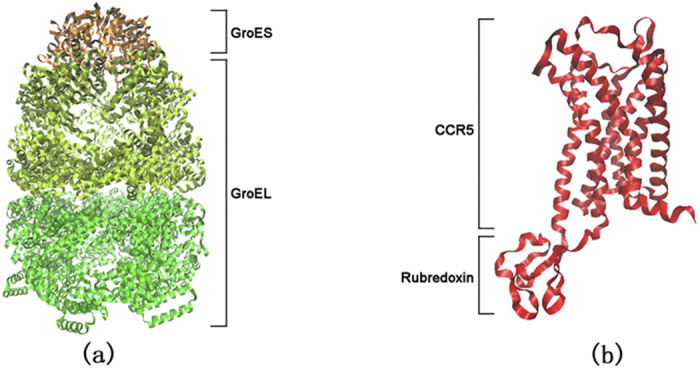
Structures of the GroEL-GroES chaperonin complex and CCR5-rubredoxin. (**a**) GroEL consists of fourteen identical subunits arranged in a pair of seven-membered rings that are stacked back-to-back to form a double doughnut-like cylindrical structure, and GroES consists of seven identical subunits arranged to form a domed disk (PDB 1SVT)^25^. The two rings of GroEL are shown in green and yellow, and GroES is shown in orange. (**b**) The CCR5 chemokine receptor consists of seven hydrophobic transmembrane helices separated by alternating intracellular and extracellular loop regions (PDB 4MBS)^36^. The diagrams of GroEL-GroES and CCR5-rubredoxin are rendered at different scales.

**Table 1:** Kinetic parameters describing the cell-free translation and folding of CCR5, its proteolysis by subtilisin and its ligand-binding capacity with or without the addition of chaperonins and/or their inhibitor DMPHBA.

Events	Kinetic parameters	No addition	DMPHBA	GroEL-GroES	GroEL-GroES+DMPHBA	GroEL
Translation	k_trans_/min^−1^	(3.0 ± 0.8) × 10^−2^	(4.6 ± 0.9) × 10^−2^	(5.2 ± 1.0) × 10^−2^	(3.6 ± 0.7) × 10^−2^	(4.1 ± 0.8) × 10^−2^
Folding	k_fold_/min^−1^	(8.2 ± 1.2) × 10^−3^	(9.6 ± 1.6) × 10^−3^	(3.0 ± 0.5) × 10^−1^	(9.1 ± 1.5) × 10^−3^	(7.0 ± 1.2) × 10^−2^
Proteolysis	k_pro_^1^/min^−1^	2.8 ± 1.2	N/D	3.9 ± 2.0	N/D	2.3 ± 1.0
	k_pro_^2^/min^−1^	(4.3 ± 0.8) × 10^−2^	N/D	(1.2 ± 0.2) × 10^−2^	N/D	(2.3 ± 0.5) × 10^−2^
Ligand binding	k_a_/M^−1^s^−1^	(4.7 ± 1.0) × 10^3^	N/D	(4.5 ± 0.8) × 10^3^	N/D	(4.6 ± 0.9) × 10^3^
	k_d_/s^−1^	(4.6 ± 1.0) × 10^−4^	N/D	(2.0 ± 0.4) × 10^−4^	N/D	(2.5 ± 0.5) × 10^−4^
	K_D_/M	(9.7 ± 0.4) × 10^−8^	N/D	(4.4 ± 0.2) × 10^−8^	N/D	(5.5 ± 0.2) × 10^−8^

N/D-not determined.
