# Supplementary material for: Folding of newly translated membrane protein CCR5 is assisted by the chaperonin GroEL-GroES
Source: Sci Rep. 2015 Nov 20;5:17037. doi: 10.1038/srep17037 (PMC4653635; doi:10.1038/srep17037)
Supplement: Supplementary Information [file srep17037-s1.docx]

**Supplementary Information**

**Folding of newly translated membrane protein CCR5 is assisted by the chaperonin GroEL-GroES**

Haixia Chi^†^, Xiaoqiang Wang^†,*^, Jiqiang Li, Hao Ren, Fang Huang^*^

*State Key* *Laboratory of Heavy Oil Processing and Center for Bioengineering and Biotechnology, China University of Petroleum (East China), Qingdao 266580, P. R. China*

^†^These authors contributed equally

^*^To whom correspondence may be addressed: wangxq001@upc.edu.cn; fhuang@upc.edu.cn

Tel: 0086-532-86981560, FAX: 0086-532-86981560

**Supplementary Figures**

**
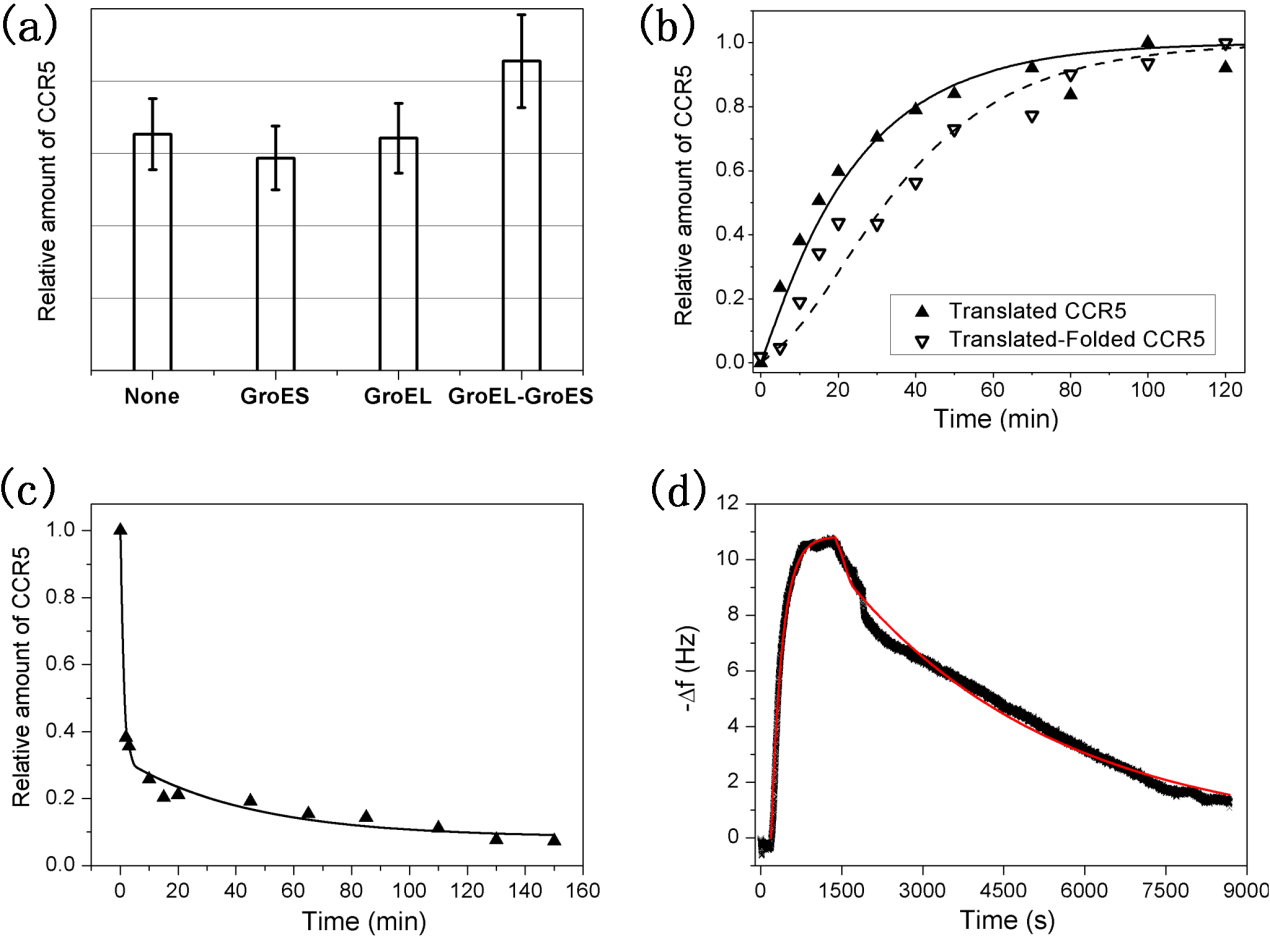
**

**Supplementary Fig. 1.** Effect of GroEL alone on the folding of CCR5. (a) Relative amounts of soluble CCR5 expressed in the cell-free system supplemented with no chaperone, GroES alone, GroEL alone or the complete chaperonin system. Averages±SD from at least three independent experiments are shown. (b) Kinetics of translation and folding of CCR5. Representative time courses for the appearance of translated and folded CCR5 were characterized in the cell-free reaction mixture supplemented with GroEL alone. Fit of the kinetic data to a single exponential equation ($F(t)=1-e^{-kt}$) is also shown. (c) Proteolysis kinetics of CCR5 by subtilisin. Representative time course for the proteolysis of CCR5 produced with only GroEL added in the cell-free system. Fit of the kinetic data to a 2-phase exponential equation ($F(t)=A_{1}*e^{-k_{1}t}+A_{2}*e^{-k_{2}t}+F_{0}$) is also shown. (d) QCM sensorgram for the binding of CCR5 to its ligand eotaxin. Representative time course that has visible association and dissociation phases is shown for CCR5 samples produced with only GroEL added in the cell-free system. Fit of the kinetic data to a 1:1 binding model is also shown.

**

**

**Supplementary Fig. 2.** Immunoblotting quantification by densitometry analysis was performed within the linear range of purified His-tagged CCR3 (also a GPCR protein) standards of known concentration expressed in mammalian HEK293 cells.





**Supplementary Fig. 3.** QCM sensorgram for the interaction of CCR5 and albumin. In the ligand flow process, albumin instead of eotaxin was used to test non-specific protein binding.
